# Nivolumab-induced capillary leak syndrome associated with chylothorax in a melanoma patient: A case report and review of the literature

**DOI:** 10.3389/fonc.2022.1032844

**Published:** 2022-12-12

**Authors:** Carole Neuville, François Aubin, Eve Puzenat, Dragos Popescu, Thomas Crepin, Charlée Nardin

**Affiliations:** ^1^ Department of Dermatology, University Hospital, Besançon, France; ^2^ Univ. Bourgogne Franche-Comté, INSERM, EFS BFC, UMR1098, RIGHT Interactions Greffon-Hôte Tumeur/Ingénierie Cellulaire et Génique, Besançon, France; ^3^ Department of Nephrology, University Hospital, Besançon, France

**Keywords:** capillary leak syndrome, chylothorax, immune checkpoint inhibitor, anti-PD1, adverse event, VEGF

## Abstract

**Introduction:**

Adverse events (AEs) of immune checkpoint inhibitors (ICIs) are frequent and mainly due to an overactivity of the immune system leading to excessive inflammatory responses (immune-related AE) that can affect any organ of the body. Beside the most frequent AEs, there are rare AEs whose diagnosis and treatment can be challenging. We report here a singular case of capillary leak syndrome (CLS) associated with chylothorax occurring in a patient who has been treated with adjuvant nivolumab (anti-PD1) for resected AJCC stage IIB primary melanoma.

**Case presentation:**

A 43-year-old woman was diagnosed with a nodular stage IIB melanoma of her left thigh, according to the AJCC 8th edition (T3bN0M0). The woman was treated with adjuvant nivolumab. She stopped the treatment after 4 infusions due to thrombopenia. Three months later, she developed facial and leg edema and ascites due to capillary leak syndrome. The CLS was associated with chylothorax and elevated vascular endothelial growth factor. The patient was initially treated with several pleural puncturing and steroids. CLS and chylothorax progressively decreased with intravenous immunoglobulins and fat-free diet without recurrence of melanoma at one-year follow-up.

**Conclusion:**

CLS is a rare and potentially life-threatening AE of ICIs such as anti-PD1. This AE may be associated with chylothorax probably related to lymphatic permeability induced by anti-PD1.

## Introduction

Immune checkpoint inhibitors (ICIs) such as anti-programmed cell death 1 (anti-PD1) and anti-cytotoxic T-lymphocyte-associated protein 4 (anti-CTLA-4) have revolutionized the prognosis of cancer. Adverse events (AEs) of ICIs are frequent and mainly due to an overactivity of the immune system leading to excessive inflammatory responses (immune-related AE) that can affect any organ of the body. Beside the most frequent AEs, such as thyroid, cutaneous, gastro-intestinal and hepatic AEs, there are rare AEs whose diagnosis and treatment can be challenging. We report here a singular case of capillary leak syndrome (CLS) associated with chylothorax occurring in a patient who had been treated with adjuvant nivolumab (anti-PD1) for resected AJCC stage IIB primary melanoma.

## Case report

**Figure 1:**
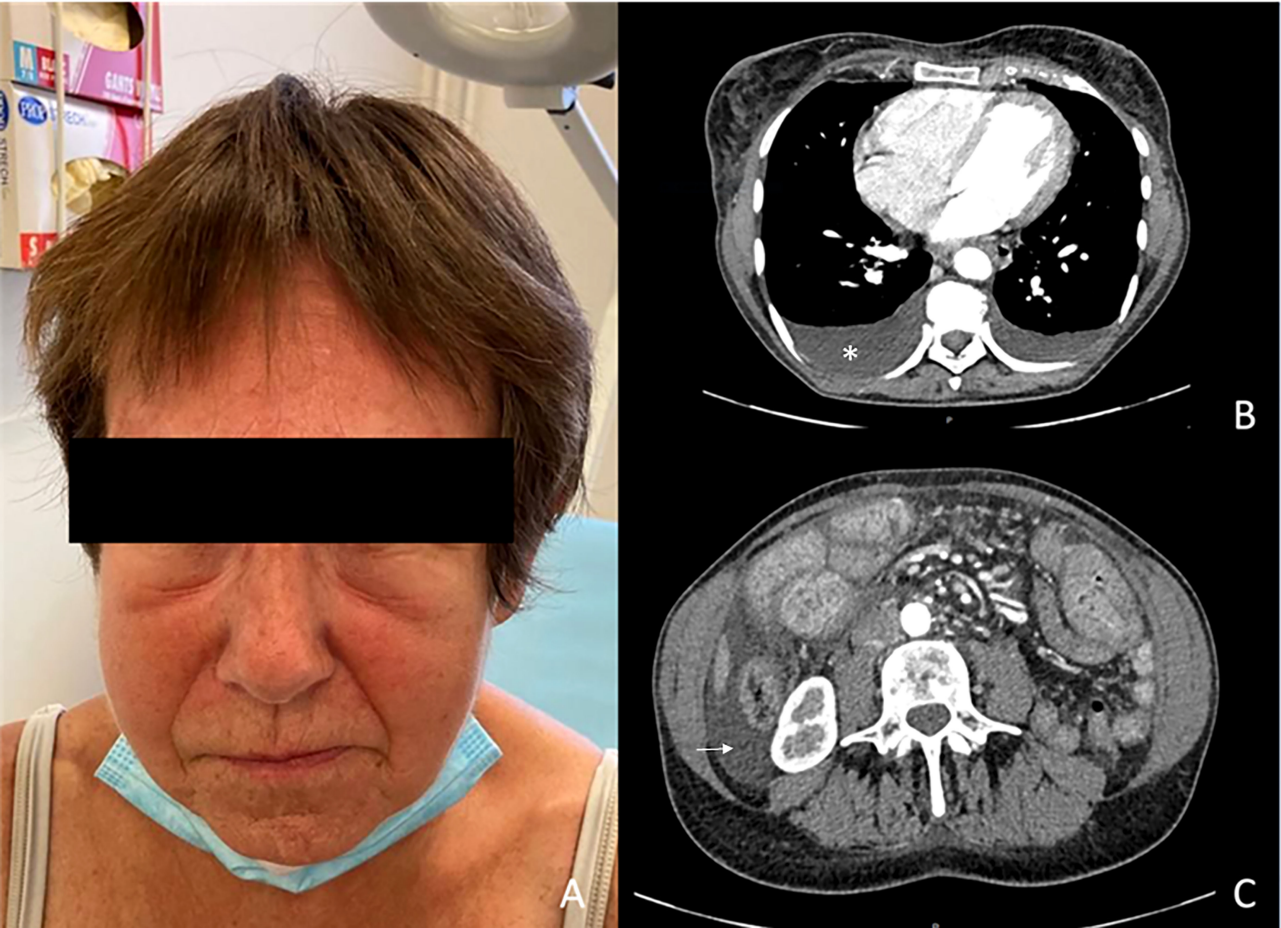
Clinical photography **(A)** and CT-scan images **(B, C)**. **(A)** Facial edema **(B)** Pleural effusion (white star). **(C)** Ascites (white arrow).

A 43-year-old woman was diagnosed with a nodular stage IIB NRAS-mutated melanoma of her left thigh, according to the AJCC 8th edition (T3bN0M0). She first underwent a wide resection of the primary lesion and started infusions of anti-PD1 antibodies (nivolumab 480 mg monthly), as part of a therapeutic trial. She developed thyroiditis after 2 infusions of nivolumab with successive phases of hyper- and then hypothyroidism which was treated with thyroid substitution. The occurrence of a grade III immune thrombocytopenia (platelets count: 27 000/mm^3^ without autoantibodies) subsequently led to the interruption of nivolumab after 4 infusions and was resolved with systemic corticosteroids (1mg/kg) and eltrombopag olamine (75mg daily). Three months after treatment discontinuation (7 months after initiation of nivolumab), she developed edema of the legs and face, along with a weight gain (+ 8 kgs), asthenia, dyspnea, and cough. Laboratory tests showed a drop of serum-albumin levels from 40g/l to 23g/l, normal hematocrit count without hemodynamics disturbances (122/69mmHg, 100 bpm), without signs of enteropathy, neither heart, kidney or liver failure. All hormonal tests (TSH, cortisol) and serum protein electrophoresis were normal. There was no proteinuria and urinalysis results were normal. The CT-scan revealed mild bilateral pleural effusion and mild ascites due to anasarca (**Figure 1**). Echocardiography also found a slight pericardial effusion without cardiopathy. Colonoscopy was normal. A scintigraphy with marked albumin (99mTc) showed a capillary hyperpermeability (Landi’s test). Thoracentesis allowed a 600cc fluid evacuation. Cytology was normal but triglycerides’ levels were elevated at 25g/L (N<1,1 g/L) in favor of chylothorax with normal levels of triglycerides in blood.

The diagnosis of a secondary form of capillary leak syndrome (CLS) with chylothorax induced by nivolumab was made in the absence of infection, vaccine and other inducing drugs. Cytokines levels were as follows: normal IL-6 levels (<3,6pg/ml) and elevated vascular endothelial growth factor (VEGF) levels (109 pg/ml, N<60 pg/ml).

High doses of corticosteroids (1mg/kg) and intravenous immunoglobulins (IVIG) (0.4 g/kg for 5 days) followed by IVIG (1g/kg/day for two consecutive days monthly) for 5 months, along with a fat-free diet and iterative pleural puncturing provided the resolution of the edema, the chylothorax and the ascites. At last follow-up (one year after nivolumab discontinuation), there was no evidence of melanoma progression according to CT-scan.

## Discussion

CLS is a very rare and potentially life-threatening AE that has been described with different treatments (1, 2).

5 cases of CLS have recently been reported with ICI, particularly anti-PD1 antibodies (nivolumab and pembrolizumab) (3–6).

In the current case, the role of eltrombopag olamine cannot be excluded even if it has never been previously reported.

This case is singular because these rare toxicities (CLS and chylothorax) occurred many months after nivolumab discontinuation. Immune-related adverse events (irAE) can be delayed and occured after the completion of ICI (> 90 days) (7). Indeed, CLS were mostly reported early during anti-PD1 treatment (5)) or soon after their discontinuation (one month after discontinuation of pembrolizumab and nivolumab) (3, 4).

This is the first case of CLS and chylothorax induced by adjuvant nivolumab in a patient with early-stage cancer (resected primary stage IIB melanoma). Until now, CLS induced by ICI has only been reported in patients with advanced cancers. Furthermore, the association of chylothorax with CLS after anti-PD1 treatment has been scarcely reported in the literature. A patient with a stage IV melanoma treated with pembrolizumab and injections of talimogene laherparepvec developed the association of CLS and lymphatic dysfunction with chylous pleural and abdominal effusions (3). Another patient died of a chylothorax related to tumor progression 12 months after initiation of nivolumab for a metastatic pulmonary adenocarcinoma (8). Thus, the association of these two adverse events does not appear coincidental and appear to be irAEs due to the same mechanisms.

In our practice, we lack parameters to identify patients at risk of severe toxicities. In the future, we will evaluate the probability of response and the risk of toxicity to evaluate the benefice/risk balance of ICI treatment, particularly with patients treated at early stage (such as this patient with resected stage II melanoma). Indeed, adjuvant pembrolizumab treatment has demonstrated its efficacy by decreasing the risk of melanoma recurrence in patients with resected stage IIB or IIC melanoma (9). However, there are frequent irAEs including severe irAEs and chronic irAEs (such as endocrine AE reported at 25%). Thus, the benefice/risk balance of ICI treatment should be discussed with the patient.

It will be necessary to stratify the treatment according predictive factors of response such as the TMB, IFN-γ-signature (10) and predictive factors of toxicity such as the fecal microbiote (11) or the diversity of the TCR clones (12).

Regarding toxicity, there are studies evaluating clinical and biological factors associated with toxicities (NCT04871542) and evaluating strategy to avoid treatment toxicity such as fecal microbiota transplantation (NCT04163289). In a phase I study, the role of microbiome modification in preventing immune-related toxicities by adding fecal microbiota transplantation to ICI therapy was associated with a safety profile in unselected metastatic renal cell carcinoma and promising clinical efficacy data (13).

However, most of these biomarkers are not performed in clinical practice (TMB, IFN-γ-signature, TCR clones) and were not available for this patient. Furthermore, there is no tool validated in clinical practice and no recommendation to predict toxicities, prevent toxicities, adapt the treatment according to the risk of toxicities and the probability of response. Therefore, these parameters cannot be use for treatment decision. If predictive factors of response and toxicities are identified and validated in prospective studies, the treatment could be adapted to increase treatment efficacy and avoid toxicity. Thus, further studies are needed to develop individualized approaches to avoid treatments toxicities.

It is remarkable to notice that CLS mainly occurred in patients with a controlled disease suggesting that this AE is associated with a strong anti-tumor immune response as reported with other AEs (14). Although the pathophysiology of CLS is not clear, T-cell activation and the release of cytokines induced by ICIs may be involved. T-CD8 cells surrounding endothelial cells in CLS have been indeed described (15).

The association of CLS with chylothorax suggests a severe endothelial dysfunction, both vascular and lymphatic, induced by ICI, involving the crosstalk between immune cells and endothelial cells (16, 17). It is known that ICI stimulate cytokines secretion by immune cells and it was reported that circulating cytokines levels (including VEGF during treatment with ICI) were increased in patients with severe irAE (18) as found in our case. This supports that the mechanisms of nivolumab-induced CLS and chylothorax involve immunity.

PD-L1 expression was found on endothelial cells and involved in T-cell mediated myocardial injury (19). It is well known that angiogenesis and immunosuppression occurs simultaneously and that there are interactions between angiogenesis and the immune response (17, 20). VEGF promotes the recruitment and proliferation of immunosuppressive cells such as Treg cells, MDSCs, and M2-TAMs, creating a more immunosuppressive environment (21). Indeed, vascular normalization may enhance antitumor immunity and lymphocyte-mediated cancer immunotherapy with immune checkpoint inhibitors (21, 22).

Furthermore, increased levels of VEGF have been reported in CLS and support the hypothesis of endothelial activation and the use of anti-VEGF therapy as found in our patient (23).

Targeting angiogenesis may increase tumor control of cancer such as melanoma (24). Indeed, the combination of anti-angiogenic therapy to ICI can potentiate anti-tumor immune response by regulating the interactions between angiogenesis and the immune response (25, 26). The combination of bevacizumab and ipilimumab was safely administered in patients with metastatic melanoma (27). Finally, strategies combining anti-angiogenic and anti-PD1 agents have been studied and appears to tip the balance of the tumor microenvironment and improve treatment response in advanced melanoma (28, 29). These results support the efficacy of anti-angiogenic agents with ICI and further investigation.

In conclusion, physicians should be aware of the possibility of a CLS associated with lymphatic permeability induced by anti-PD1. Despite the favorable melanoma prognosis, this IRAE may be life-threatening.

## Data availability statement

The raw data supporting the conclusions of this article will be made available by the authors, without undue reservation.

## Ethics statement

Ethical review and approval was not required for the study on human participants in accordance with the local legislation and institutional requirements. The patients/participants provided their written informed consent to participate in this study. Written informed consent was obtained from the individual(s) for the publication of any potentially identifiable images or data included in this article.

## Author contributions

Substantial contributions to conception and design: ChN, CaN, EP, DP, TC, FA. Acquisition of data: ChN, CaN, EP, DP. Analysis and interpretation of data: ChN, TC, FA. Drafting the article: ChN, CaN, EP and DP. Revising the article: ChN, TC, FA. Final approval of the version to be published: CaN, ChN, EP, DP, TC, FA. All authors contributed to the article and approved the submitted version.
